# Effectiveness and safety in non-valvular atrial fibrillation patients switching from warfarin to direct oral anticoagulants in US healthcare claims

**DOI:** 10.1007/s11239-024-02976-1

**Published:** 2024-05-02

**Authors:** Gregory Y. H. Lip, Virginia Noxon, Amiee Kang, Xuemei Luo, Nipun Atreja, Stella Han, Dong Cheng, Jenny Jiang, Lisa Abramovitz, Steven Deitelzweig

**Affiliations:** 1 Liverpool Centre for Cardiovascular Science, University of Liverpool, Liverpool John Moores University and Liverpool Heart and Chest Hospital, Liverpool, UK; 2 Danish Center for Clinical Health Services Research, Department of Clinical Medicine, Aalborg University, Aalborg, Denmark; 3 STATinMED LLC, Dallas, TX USA; 4 Bristol-Myers Squibb Company, New York, NY USA; 5 Pfizer Inc, New York, NY USA; 6 Department of Hospital Medicine, Ochsner Clinic Foundation, New Orleans, LA USA; 7 Faculty of Health and Life Sciences, University of Liverpool, Foundation Building, Brownlow Hill, Liverpool, L69 7TX UK

**Keywords:** Stroke/systemic embolism, Non-valvular atrial fibrillation, Direct oral anticoagulant, Warfarin, Switching, Apixaban

## Abstract

**Introduction:**

There is a paucity of real-world studies examining the risks of stroke/systemic embolism (SE) and major bleeding (MB) among non-valvular atrial fibrillation (NVAF) patients switching from warfarin to a direct oral anticoagulant (DOAC). This retrospective study was conducted to compare the stroke/SE and MB risks between patients switched from warfarin to apixaban, dabigatran, or rivaroxaban in real-world clinical practice.

**Materials and methods:**

This study used data from four United States commercial claims databases from January 1, 2012 to June 30, 2019. The study population included NVAF patients initially treated with warfarin and switched to apixaban, dabigatran, or rivaroxaban within 90 days of their warfarin prescription ending. Patients were matched 1:1 between the DOACs in each database using propensity scores and then pooled for the final analysis. Cox proportional hazards models were used to calculate the risk of stroke/SE and MB.

**Results and conclusions:**

The final population consisted of 2,611 apixaban-dabigatran, 12,165 apixaban-rivaroxaban, and 2,672 dabigatran-rivaroxaban pairs. Apixaban vs. dabigatran was associated with a lower risk of stroke/SE (hazard ratio [HR]: 0.61; 95% confidence interval [CI]: 0.39–0.96) and MB (HR: 0.67; 95% CI: 0.50–0.91). Apixaban vs. rivaroxaban was associated with a similar risk of stroke/SE (HR: 0.88; 95% CI: 0.73–1.07) and a lower risk of MB (HR: 0.60; 95% CI: 0.52–0.68). There was no significant difference in either risk between dabigatran and rivaroxaban. These results provide important insights into how the risks of stroke/SE and MB for NVAF patients vary when switching from warfarin to different DOACs.

**Supplementary Information:**

The online version contains supplementary material available at 10.1007/s11239-024-02976-1.

## Key point

• Patients with NVAF may switch to DOACs for effectiveness, safety, or convenience.

• Risk of stroke/SE and MB may vary among patients with NVAF who switch to DOACs.

• Switching to apixaban had a lower risk of MB than dabigatran/rivaroxaban.

• Results may inform DOAC prescribing decisions after warfarin in patients with NVAF.

**Supplementary Information:**

The online version contains supplementary material available at 10.1007/s11239-024-02976-1.

## Introduction

Non-valvular atrial fibrillation (NVAF) is a cardiac arrhythmia and significant cause of stroke and mortality in the United States (US) [1, 2]. Approximately 4% of NVAF patients experience stroke and around 11% die within 1 year of initial diagnosis [3]. The incidence of atrial fibrillation in the US is predicted to increase from 5.2 million cases in 2010 to 12.1 million cases in 2030 [4].

Oral anticoagulants (OACs) such as the vitamin K antagonist warfarin are typically used to prevent stroke in patients with NVAF. While vitamin K antagonists decrease the risk of stroke, there are concerns around long-term safety and increased risk of bleeding [5–11]. Vitamin K antagonists have some of the highest rates per drug class of emergency admissions to hospital in the elderly, chronic anticoagulation monitoring is needed, there are potentially life-threatening interactions with foods and other drugs, and a long period is needed for onset and offset of drug action [12, 13]. As a result of these limitations, clinicians may hesitate to prescribe warfarin for NVAF patients, and they will consequently be at an increased risk of stroke [14].

Direct-acting OACs (DOACs; including apixaban, dabigatran, edoxaban, and rivaroxaban) have provided a convenient, effective, and tolerable alternative to warfarin [13]. Compared to warfarin, DOACs can be prescribed in fixed doses and anticoagulation monitoring is not needed [15–17]. Clinical trials have also shown that DOACs have similar or better efficacy and safety vs. warfarin [18]. Due to their increased convenience, efficacy, and safety, patients may be switched from warfarin to a DOAC in clinical practice settings [19].

To ensure appropriate treatment, it is important to understand the effectiveness and safety of each DOAC in patients who switch from warfarin [20–27]. Research to date on anticoagulant therapy for NVAF has focused on warfarin or warfarin versus DOACs, and few studies have compared the outcomes between different DOACs in patients switching from warfarin [28, 29]. Given that pharmacokinetic differences between DOACs may affect their effectiveness and safety, this is a significant gap in the research. To try to fill this gap, this study aimed to compare stroke/systemic embolism (SE) and MB outcomes in NVAF patients who switched from warfarin to different DOACs in the real-world setting.

## Materials and methods

### Data source

Data were collected and pooled from the following four US commercial claims databases: IQVIA LifeLink PharMetrics Plus, Truven MarketScan Commercial Claims and Encounters, OptumInsight, and the Humana database. IQVIA LifeLink PharMetrics Plus is a claims database for medical (provider and institutional) and pharmacy services in the US covering around 40 million lives per year. Truven MarketScan is a combined claims database of employer- and health-plan-sourced data containing medical and drug data for several million individuals annually, including over 94 million unique patients since 1996. OptumInsight serves > 125 million individuals; OptumInsight is a proprietary research database containing claims and enrollment data dating back to 1993. Finally, Humana includes more than 11.3 million lives of commercial and Medicare members covering all census regions of the US. The study period was January 2012 to March 2019 for the IQVIA LifeLink PharMetrics Plus, OptumInsight, and Humana databases, and January 2012 to June 2019 for the Truven MarketScan database. The identification period was 1-Jan-2013 through 30-Jun-2019 in all databases.

### Patient selection

Patients from each database with an AF diagnosis (ICD-9-CM code of 427.31; ICD-10-CM code I480-I482, I4891) based on International Classification of Diseases 9th and 10th revisions, Clinical Modification (ICD-9-CM/ICD-10-CM) codes between 01-Jan-2012 and 30-Jun-2019, and at least one pharmacy claim for warfarin, apixaban, dabigatran, rivaroxaban, or edoxaban during the identification period were selected. Among these patients, all OAC treatment episodes were identified. OAC treatment episodes were defined as the time from the date of the first prescription for a specific OAC to the earliest of OAC discontinuation, treatment switch, end of the study period, and death or disenrollment. Treatment episodes were eligible for the study if patients were aged ≥ 18 years on the OAC prescription date, had continuous health enrollment with medical and pharmacy benefits for ≥ 12 months prior to and on the OAC prescription date, and had ≥ 1 medical claim for AF prior to or on the OAC prescription date. Treatment episodes were excluded if there were medical claims indicating a diagnosis or procedure for rheumatic mitral valvular heart disease, valve replacement procedure, or venous thromboembolism during the 12 months prior to or on the OAC prescription date, pregnancy during the study period, diagnosis or procedure for transient AF (heart valve replacement/transplant, pericarditis, hyperthyroidism, thyrotoxicity) during the 12 months prior to or on the OAC prescription date, or hip/knee replacement surgery within 6 weeks prior to the OAC prescription date. Treatment episodes with no follow-up were also excluded.

Among patients whose OAC treatment episodes were all eligible for inclusion, those with a DOAC treatment episode within 90 days of a warfarin treatment episode were finally selected for the study. Time between warfarin episode end date and first DOAC start date was considered. Patients from each database were divided into four treatment groups based on their first DOAC prescription after warfarin (apixaban, dabigatran, edoxaban, or rivaroxaban). The date of first DOAC prescription was the index date. The baseline period was 12 months prior to and including the index date for all cohorts. Patients from all databases were pooled and followed from the day after the index date until death, the end of the study period, discontinuation, another treatment switch, or the end of continuous medical and pharmacy enrollment, whichever occurred first. Discontinuation was defined as no evidence of a prescription for the index DOAC for 30 days from the last day’s supply of the last filled prescription plus 30 days. The date of discontinuation was defined as the last day of days’ supply of last filled prescription. NVAF patients who received a prescription for an OAC (warfarin, apixaban, dabigatran, edoxaban, or rivaroxaban) other than the index drug prescription during the follow-up period were considered switchers if this OAC prescription was within 30 days of the last day’s supply.

### Outcome measures

The primary effectiveness outcome was stroke/SE and the primary safety outcome was MB. Stroke/SE event was defined as an acute-care inpatient admission with a corresponding primary or first listed ICD-9-CM or ICD-10-CM diagnosis or procedure code at any time during follow-up. Hemorrhagic Stroke, Ischemic Stroke and Systemic Embolism are considered under Stroke/SE. MB was defined as an acute-care inpatient admission with a corresponding primary or first listed ICD-9-CM or ICD-10-CM diagnosis or procedure code at any time during follow-up. Major Gastrointestinal bleeding event, Major Intracranial Hemorrhage (ICH) and Major Other hemorrhage are considered under MB.

### Statistical analysis

Baseline demographics, clinical characteristics, and comorbidities were summarized separately for patients switching to apixaban, dabigatran, edoxaban, and rivaroxaban. Race is not available in all of the commercial claim’s databases considered for this study, so it was not included as a patient characteristic. Baseline medication use if determined by at least one prescription filled for specific drugs. The CHA2DS2-VASc score is based on following characteristic: congestive heart failure, hypertension, age ≥75 (doubled), diabetes mellitus, prior stroke or transient ischemic attack (doubled), vascular disease, age 65–74, female. CHADS2 score is based on following characteristics: congestive heart failure, hypertension, age ≥75 years, diabetes mellitus and stroke. HAS-BLED is based on Hypertension, Abnormal Renal/Liver Function, Stroke, Bleeding History or Predisposition, Labile INR, Elderly, Drugs/Alcohol. Mean, standard deviation (SD), median, and interquartile range (IQR) were used to describe continuous variables. Differences across treatments were compared using the student’s t-test or Wilcoxon rank sum test. Percentages were presented for categorical and binary variables and compared using the chi-square test or Fishers Exact test. One-to-one propensity score matching (PSM) was used to adjust for differences in baseline characteristics. Patients were matched within the same database to account for heterogeneity across datasets. Propensity score was defined as the probability of being treated with each DOAC based on a set of baseline characteristics in the DOAC cohort. Propensity scores were estimated using unconditional logistic regression analyses incorporating baseline characteristics as independent variables in the regression and status of each DOAC as the outcome. The nearest neighbor method without replacement and with a caliper of 0.01 was used to select matched samples.

All baseline variables were evaluated as covariates to be included in multivariate models. Stroke/SE was stratified into ischemic stroke, hemorrhagic stroke, and SE. MB events were stratified into gastrointestinal (GI) bleeding, intracranial hemorrhage (ICH), and other bleeding. Time to stroke/SE and MB events was calculated from the day after the index date to the date of the event. Incidence rates were calculated per 100 person-years, with the numerator being the number of patients with an event and the denominator the time at risk.

Cox proportional hazards models were used to compare the time to MB and stroke between apixaban versus dabigatran, apixaban versus rivaroxaban, and dabigatran versus rivaroxaban. The proportional hazards proportionality assumption was evaluated by visually inspecting the Kaplan-Meier plot within the matched cohorts and confirmed by testing the significance of interactions between treatment and the log of time. If this assumption was invalidated, the addition of an interaction term of time or time-dependent covariate was added.

## Results

### Patient characteristics

**Fig. 1:**
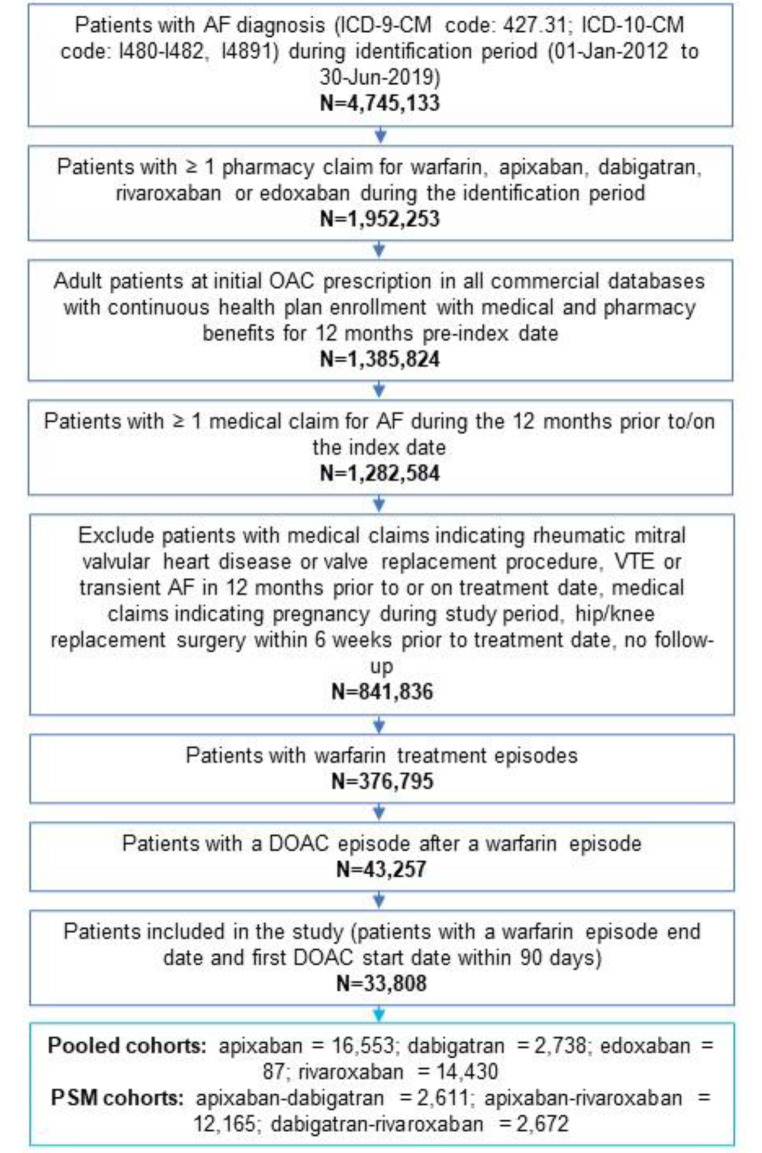
Patient selection criteria *AF* atrial fibrillation, *DOAC* direct-acting oral anticoagulation, *ICD-10-CM* International Classification of Diseases, Tenth Revision, Clinical Modification, *ICD-9-CM* International Classification of Diseases, Ninth Revision, Clinical Modification, *OAC* oral anticoagulation, *PSM* propensity score matched, *VTE* venous thromboembolism.

Figure 1 shows the patient selection process. A total of 376,795 patients across the four databases underwent warfarin treatment during the study identification period and met the other study inclusion and exclusion criteria. Of these, 33,808 patients had DOAC treatment within 90 days of warfarin discontinuation and were eligible for the analysis: 16,553 patients were in the apixaban cohort, 2,738 in the dabigatran cohort, 14,430 in the rivaroxaban cohort, and 87 in the edoxaban cohort (Fig. 1). PSM was not conducted for edoxaban patients due to the small sample size. After PSM, there were 2,611-2,611 apixaban-dabigatran, 12,165 − 12,165 apixaban-rivaroxaban, and 2,672-2,672 dabigatran-rivaroxaban matched pairs (Table 1).

**Table 1:** Propensity score-matched pooled baseline characteristics of patients switched from warfarin to apixaban, dabigatran, or rivaroxaban

	Apixaban cohort (*n* = 2,611)	Dabigatran cohort (reference)		Apixaban cohort (*n* = 12,165)	Rivaroxaban cohort (reference)		Dabigatran cohort (*n* = 2,672)	Rivaroxaban cohort (reference)		
	N/mean (%/SD)	N/mean (%/SD)	STD^a^	N/mean (%/SD)	N/mean (%/SD)	STD^a^	N/mean (%/SD)	N/mean (%/SD)		STD
Age, years	70.61 (12.5)	71.1 (12.5)	3.8	72.2 (12.1)	72.2 (12.2)	0.1	70.8 (12.6)	70.4 (12.2)		3.4
18–54	211 (8.1%)	204 (7.8%)	1.0	786 (6.5%)	775 (6.4%)	0.4	223 (8.4%)	216 (8.1%)		1.00
55–64	642 (24.6%)	645 (24.7%)	0.3	2,500 (20.6%)	2,510 (20.6%)	0.2	681 (25.5%)	696 (26.1%)		1.3
65–74	726 (27.8%)	709 (27.2%)	1.5	3,433 (28.2%)	3,478 (28.6%)	0.8	711 (26.6%)	753 (28.2%)		3.5
75–79	472 (18.1%)	472 (18.1%)	0.00	2,370 (19.5%)	2,359 (19.4%)	0.2	472 (17.7%)	446 (16.7%)		2.6
≥ 80	560 (21.5%)	581 (22.3%)	2.0	3,076 (25.3%)	3,043 (25.0%)	0.6	585 (21.9%)	561 (21.0%)		2.2
Gender										
Male	1,542 (59.1%)	1,558 (59.7%)	1.3	6,904 (56.8%)	6,913 (56.8%)	0.2	1,597 (59.8%)	1,557 (58.3%)		3.0
Female	1,069 (40.9%)	1,053 (40.3%)	1.3	5,261 (43.3%)	5,252 (43.2%)	0.2	1,075 (40.2%)	1,115 (41.7%)		3.0
US geographic region										
Northeast	422 (16.2%)	419 (16.1%)	0.3	1,684 (13.8%)	1,675 (13.8%)	0.2	433 (16.2%)	445 (16.7%)		1.2
Midwest	546 (20.9%)	541 (20.7%)	0.3	2,733 (22.5%)	2,688 (22.1%)	1.1	557 (20.9%)	550 (20.6%)		0.7
South	1,078 (41.3%)	1,080 (41.4%)	0.2	5,072 (41.7%)	5,117 (42.1%)	0.8	1,085 (40.6%)	1,100 (41.2%)		1.1
West	562 (21.5%)	559 (21.4%)	0.3	2,632 (21.6%)	2,638 (21.7%)	0.1	582 (21.8%)	563 (21.1%)		1.7
Other	3 (0.11%)	12 (0.5%)	6.4	44 (0.4%)	47 (0.4%)	0.4	15 (0.6%)	14 (0.5%)		0.5
Baseline comorbidity										
Deyo-Charlson Comorbidity Index	2.8 (2.6)	2.84 (2.7)	0.1	3.15 (2.7)	3.2 (2.8)	0.4	2.78 (2.6)	2.77 (2.6)		0.4
CHADS_2_ score	2.4 (1.4)	2.50 (1.4)	4.6	2.52 (1.4)	2.6 (1.4)	2.5	2.48 (1.4)	2.42 (1.4)		4.1
0	123 (4.7%)	142 (5.4%)	3.3	520 (4.3%)	540 (4.4%)	0.8	151 (5.7%)	148 (5.5%)		0.5
1	577 (22.1%)	512 (19.6%)	6.1	2,338 (19.2%)	2,375 (19.5%)	0.8	530 (19.8%)	591 (22.1%)		5.6
2	790 (30.3%)	778 (29.8%)	1.0	3,702 (30.4%)	3,498 (28.8%)	3.7	800 (29.9%)	785 (29.4%)		1.2
3+	1,121 (42.9%)	1,179 (45.2%)	4.5	5,605 (46.1%)	5,752 (47.3%)	2.4	1,191 (44.6%)	1,148 (43.0%)		3.2
CHA_2_DS_2_-VASc score	3.82 (1.9)	3.89 (1.9)	3.6	4.01 (1.9)	4.0 (1.9)	1.9	3.84 (1.9)	3.8 (1.9)		2.7
0	59 (2.3%)	71 (2.7%)	3.0	229 (1.9%)	261 (2.2%)	1.9	77 (2.9%)	70 (2.6%)		1.6
1	239 (9.2%)	230 (8.8%)	1.2	882 (7.3%)	884 (7.3%)	0.1	245 (9.2%)	233 (8.7%)		1.6
2	403 (15.4%)	381 (14.6%)	2.4	1,564 (12.9%)	1,551 (12.8%)	0.3	401 (15.0%)	426 (15.9%)		2.6
3	474 (18.2%)	449 (17.2%)	2.5	2,207 (18.1%)	2,162 (17.8%)	1.0	459 (17.2%)	504 (18.9%)		4.4
4+	1,436 (55.0%)	1,480 (56.7%)	3.4	7,283 (59.9%)	7,307 (60.1%)	0.4	1,490 (55.8%)	1,439 (53.9%)		3.8
HAS-BLED score	2.86 (1.4)	2.84 (1.4)	1.0	2.95 (1.4)	2.96 (1.4)	0.7	2.81 (1.4)	2.8 (1.4)		0.2
0	67 (2.6%)	96.00 (3.7%)	6.4	291 (2.4%)	314.00 (2.6%)	1.2	106 (4.0%)	90.0 (3.4%)		3.2
1	370 (14.2%)	366 (14.0%)	0.4	1,415 (11.6%)	1,396 (11.5%)	0.5	390 (14.6%)	379 (14.2%)		1.2
2	652 (25.0%)	628 (24.1%)	2.1	2,981 (24.5%)	2,918 (24.0%)	1.2	646 (24.2%)	677 (25.3%)		2.7
3+	1,522 (58.3%)	1,521 (58.3%)	0.1	7,478 (61.5%)	7,537 (62.0%)	1.0	1,530 (57.3%)	1,526 (57.1%)		0.3
Bleeding history	669 (25.6%)	640 (24.5%)	2.6	3,051 (25.1%)	3,041 (25.0%)	0.2	644 (24.1%)	649 (24.3%)		0.4
CHF	856 (32.8%)	850 (32.6%)	0.5	4,255 (35.0%)	4,244 (34.9%)	0.2	855 (32.0%)	843 (31.6%)		1.0
Diabetes mellitus	1,048 (40.1%)	1,064 (40.8%)	1.3	5,005 (41.1%)	5,054 (41.6%)	0.8	1,093 (40.9%)	1,102 (41.2%)		0.7
Hypertension	2,322 (88.9%)	2,306 (88.3%)	1.9	10,837 (89.1%)	10,812 (88.9%)	0.7	2,351 (88.0%)	2,344 (87.7%)		0.8
Renal disease	618 (23.7%)	615 (23.6%)	0.3	3,236 (26.6%)	3,277 (26.9%)	0.8	605 (22.6%)	585 (21.9%)		1.8
Liver disease	143 (5.5%)	139 (5.3%)	0.7	674 (5.5%)	670 (5.5%)	0.1	143 (5.4%)	156 (5.8%)		2.1
Myocardial infarction	333 (12.8%)	315 (12.1%)	2.1	1,424 (11.7%)	1,429 (11.8%)	0.1	309 (11.6%)	307 (11.5%)		0.2
Dyspepsia or stomach discomfort	480 (18.4%)	479 (18.4%)	0.1	2,214 (18.2%)	2,258 (18.6%)	0.9	489 (18.3%)	481 (18.0%)		0.8
Non-stroke/ SE peripheral vascular disease	592 (22.7%)	607 (23.3%)	1.4	3,184 (26.2%)	3,177 (26.1%)	0.1	603 (22.6%)	603 (22.6%)		0.0
Stroke/SE	419 (16.1%)	405 (15.5%)	1.5	1,775 (14.6%)	1,756 (14.4%)	0.4	410 (15.3%)	400 (15.0%)		1.0
TIA	290 (11.1%)	274 (10.5%)	2.0	1,358 (11.2%)	1,364 (11.2%)	0.2	281 (10.5%)	273 (10.2%)		1.0
Anemia and coagulation defects	832 (31.9%)	766 (29.3%)	5.5	4,159 (34.2%)	3,970 (32.6%)	3.3	767 (28.7%)	807 (30.2%)		3.3
Alcoholism	77 (3.0%)	64 (2.5%)	3.1	340 (2.8%)	341 (2.8%)	0.1	72 (2.7%)	70 (2.6%)		0.5
Peripheral artery disease	552 (21.1%)	599 (22.9%)	4.3	2,996 (24.6%)	3,066 (25.2%)	1.3	596 (22.3%)	583 (21.8%)		1.2
Coronary artery disease	1,117 42.8%	1,113 (42.6%)	0.3	5,301 (43.6%)	5,231 (43.0%)	1.2	1,116 (41.8%)	1,102 (41.2%)		1.1
Baseline medication use										
ACE/ARB	1,647 (63.1%)	1,723 (66.0%)	6.1	7,581 (62.3%)	7,629 (62.7%)	0.8	1,755 (65.7%)	1,673 (62.6%)		6.4
Amiodarone	376 (14.4%)	315 (12.1%)	6.9	1,657 (13.6%)	1,397 (11.5%)	6.5	323 (12.1%)	307 (11.5%)		1.9
Beta blockers	1,608 (61.6%)	1,592 (61.0)%	1.3	7,435 (61.1%)	7,243 (59.5%)	3.2	1,627 (60.9%)	1,618 (60.6%)		0.7
H2-receptor antagonist	170 (6.5%)	175 (6.7%)	0.8	820 (6.7%)	809 (6.7%)	0.4	177 (6.6%)	173 (6.5%)		0.6
Proton pump inhibitor	758 (29.0%)	813 (31.1%)	4.6	3,821 (31.4%)	3,679 (30.2%)	2.5	826 (30.9%)	817 (30.6%)		0.7
Statins	1,701 (65.2%)	1,686 (64.6%)	1.2	8,049 (66.2%)	8,026 (66.0%)	0.4	1,722 (64.5%)	1,775 (66.4%)		4.2
Anti-platelets	260 (10.0%)	257 (9.8%)	0.4	1,220 (10.0%)	1,168 (9.6%)	1.4	259 (9.7%)	260 (9.7%)		0.1
NSAIDs	461 (17.7%)	506 (19.4%)	4.4	2,132 (17.5%)	2,251 (18.5%)	2.6	518 (19.4%)	525 (19.7%)		0.7
Dose of the index prescription										
Standard dose (5 mg apixaban, 150 mg dabigatran, 20 mg rivaroxaban)	2,123 (81.3%)	2,248 (86.1%)	13.0	9,625 (79.1%)	9,385 (77.2%)	4.8	2,310 (86.5%)	2,147 (80.4%)		16.5
Low dose (2.5 mg apixaban, 75 mg dabigatran, 15 mg rivaroxaban)	488 (18.7%)	354 (13.6%)	14.0	2,546 (20.9%)	2,428 (20.0%)	2.4	353 (13.2%)	442 (16.5%)		9.4
Other dose (rivaroxaban 10 mg, dabigatran 110 mg)	0 (0.00%)	10 (0.4%)	8.8	0 (0.0%)	375 (3.1%)	25.2	10 (0.4%)	88 (3.3%)		21.9
Events during the baseline										
Stroke/SE hospitalization	165 (6.3%)	168 (6.4%)	0.5	652 (5.4%)	640 (5.3%)	0.4	165 (6.2%)	150 (5.6%)		2.4
Major bleed hospitalization	112 (4.3%)	116 (4.4%)	0.8	489 (4.0%)	461 (3.8%)	1.2	112 (4.2%)	114 (4.3%)		0.4
Events during the 90 days before the index date										
Stroke/SE	71 (2.7%)	72 (2.8%)	0.3	322 (2.7%)	317 (2.6%)	0.3	74 (2.8%)	63 (2.4%)		3.1
Bleeding event	358 (13.7%)	327 (12.5%)	3.5	1,551 (12.8%)	1,452 (11.9%)	2.5	327 (12.2%)	314 (11.8%)		1.5
Gap length between warfarin discontinuation to NOAC initiation	9 (17.6)	7 (16.9)	7.5	9 (17.9)	8 (16.6)	7.8	7 (16.8)	7 (15.7)		2.4
Minimum	1	1		1	1		1	1		
Q1	1	1		1	1		1	1		
Median	1	1		1	1		1	1		
Q3	3	1		3	1		1	1		
Maximum	90	90		90	90		90	90		
Length of warfarin therapy	179 (209.0)	180 (210.2)	0.5	230 (264.7)	227 (266.2)	0.9	176 (204.6)	176 (200.4)		0.2
Follow-up Time (Days)	340.09	350.50	13.82	331.41	346.00	4.15	293.48	354.05		17.95

*ACE* angiotensin-converting enzyme, *ARB* angiotensin receptor blocker, *CHF* congestive heart failure, *DOAC* direct-acting oral anticoagulant, *NSAID* non-steroidal anti-inflammatory drug, *SD* standard deviation, *SE* systemic embolism, *STD* standard, *TIA* transient ischemic attack, *US* United States

^*^STD difference = 100*[actual STD difference]. STD difference greater than 10 is considered significant

Following PSM, patient demographics were balanced for each matched cohort. The average age for the apixaban-dabigatran, apixaban-rivaroxaban, and dabigatran-rivaroxaban matched cohorts was 70–72 years old. All matched cohorts were more likely to be male (57–60% across cohorts) and reside in the Southern region of the US (40–42% across cohorts). The most common baseline comorbidities among all matched cohorts were hypertension (87–89% across cohorts), diabetes mellitus (40–41% across cohorts), and coronary artery disease (41–43% across cohorts).

### Primary outcome results

**Fig. 2:**
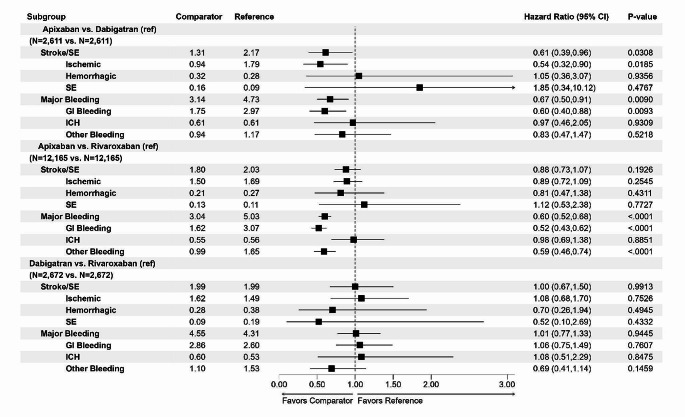
Comparison of stroke/SE and MB among different DOACs in patients switched from warfarin after PSM

After PSM, apixaban was associated with lower risks of stroke/SE and MB compared with dabigatran (stroke: 1.31 vs. 2.17, HR 0.61, 95% CI 0.39–0.96; MB: 3.14 vs. 4.73, HR 0.67, 95% CI 0.50–0.91). Within the stroke/SE category, apixaban was associated with a lower risk of ischemic stroke (0.94 vs. 1.79, HR 0.54, 95% CI 0.32–0.90), similar risk of hemorrhagic stroke (0.32 vs. 0.28, HR 1.05, 95% CI 0.36–3.07), and similar risk of SE (0.16 vs. 0.09, HR 1.85, 95% CI 0.34–10.12) when compared with dabigatran. Within the MB category, apixaban was associated with a lower risk of GI bleeding (1.75 vs. 2.97, HR 0.60, 95% CI 0.40–0.88), similar risk of ICH (0.61 vs. 0.61, HR 0.97, 95% CI 0.46–2.05), and similar risk of other bleeding when compared with dabigatran (0.94 vs. 1.17, HR 0.83; 95% CI 0.47–1.47) (Fig. 2).

When compared with rivaroxaban, apixaban was associated with a similar risk of stroke/SE (1.80 vs. 2.03, HR 0.88, 95% CI 0.73–1.07) and a lower risk of MB (3.04 vs. 5.03, HR 0.60, 95% CI 0.52–0.68). Within the stroke/SE category, apixaban was associated with similar risks of ischemic stroke (1.50 vs. 1.69, HR 0.89, 95% CI 0.72–1.09) and hemorrhagic stroke (0.21 vs. 0.27, HR 0.81, 95% CI 0.47–1.38), and a similar risk of SE as rivaroxaban (0.13 vs. 0.11, HR 1.12, 95% CI 0.53–2.38). Within the MB category, apixaban was associated with lower risks of GI (1.62 vs. 3.07, HR 0.52, 95% CI 0.43–0.62) and other bleeding (0.99 vs. 1.65, HR 0.59, 95% CI 0.46–0.74), but a similar risk of ICH (0.55 vs. 0.56, HR 0.98, 95% CI 0.69–1.38) compared with rivaroxaban (Fig. 2).

Dabigatran patients had similar risks of stroke/SE (1.99 vs. 1.99, HR 1.00, 95% CI 0.67–1.50) and MB (4.55 vs. 4.31, HR 1.01, 95% CI 0.77–1.33) compared to rivaroxaban patients (Fig. 2).

## Discussion

Using data from four large commercial healthcare claims databases, this study compared the risks of stroke/SE and MB between patients who switched from warfarin to different DOACs – apixaban, dabigatran, or rivaroxaban – in real-world clinical practice. Use of apixaban post-switch was associated with a significantly lower risk of stroke/SE when compared to dabigatran and a similar risk of stroke/SE when compared to rivaroxaban. Use of apixaban post-switch was also associated with a significantly lower risk of MB than both dabigatran and rivaroxaban. There was no significant difference between dabigatran and rivaroxaban for risk of stroke/SE or MB.

To the best of our knowledge, this is the first real-world data study comparing stroke/SE and MB outcomes among a large US sample of NVAF patients who switched from warfarin to different DOACs. Understanding the differential risks of stroke/SE and MB in NVAF patients who switch from warfarin to DOACs is of clinical importance. The findings of this analysis are generally consistent with published real-world studies that have compared stroke/SE and MB among NVAF patients who initiated different DOACs in the US as their first OAC [30, 31].

International guidelines all now recommend DOACs instead of warfarin to prevent the risk of stroke/SE for patients with AF [28, 29, 32, 33]. Consequently, NVAF patients who initiate warfarin can be switched to DOACs for legitimate clinical reasons [19]. However, different DOACs have varying efficacy and safety in patients with NVAF. Of 18,201 patients with AF and at least one additional risk factor for stroke in the ARISTOTLE clinical trial of apixaban versus warfarin, stroke/SE (HR 0.79, 95% CI 0.66–0.95) and MB (HR 0.69, 95% CI 0.60–0.80) occurred less frequently in the apixaban group than the warfarin group [23]. In the ROCKET AF clinical trial of rivaroxaban versus warfarin, of 14,264 patients with NVAF who were at increased risk for stroke, stroke/SE (HR 0.79, 95% CI 0.66–0.96) occurred less frequently in the rivaroxaban group than the warfarin group, while MB and non-major clinically relevant bleeding were similar between the rivaroxaban and warfarin group (HR 1.03, 95% CI 0.96–1.11) [34]. In the RELY clinical trial of dabigatran versus warfarin, 18,113 patients with AF and a risk of stroke, stroke/SE (110 mg dabigatran: relative risk 0.91, 95% CI 0.74–1.11; 150 mg dabigatran: relative risk 0.66, 95% CI 0.53–0.82) and MB were lower and similar in the dabigatran groups than the warfarin group [24]. Extending from the previous clinical trials that compared each DOAC with warfarin and RWD studies that compared different DOACs as their first OAC, the current study provides information on patients who initiated warfarin and later switched to different DOACs. The findings from this study can help aid clinical decisions about which DOAC may be used when patients plan to switch from warfarin to DOACs. Multiple factors can impact the decision to switch from warfarin to DOACs or which DOAC should be switched to. The decision can be influenced by medical reasons such as their history of stroke or bleeding events. Non-medical factors such as utilization management measures can also impact which DOAC patients can access. For example, in 2022, a large pharmacy benefit manager (PBM) in the US removed apixaban from Preferred Drug List, creating barriers for patients to access this medicine. After the pushbacks from patients and physicians, apixaban was reinstated on the Preferred Drug List after six month of non-coverage for some patients [35]. Patient out-of-pocket costs can also affect the utilization of DOACs [36].

This study leveraged four commercial databases in the US and included a large number of NVAF patients who switched from warfarin to DOACs. However, the study has some limitations. Due to the observational nature of this study, causal relationships cannot be determined between study variables and outcomes of interest. Potential residual confounders such as over-the-counter aspirin use, serum creatinine/creatinine clearance, and laboratory values were unavailable in the data, resulting in potential bias. Medications were based on pharmacy fills, which do not necessarily represent the patient taking the prescribed medication. Due to the nature of the data, laboratory results such as creatinine clearance or INR were not available. Patients may have been included more than once due to potential overlap between databases; however, the likelihood of duplicate observations is relatively low and so is not likely to have a significant impact on study results. Human data entry errors are possible and may lead to coding errors and misclassification of some variables. Finally, the findings of this study may not be generalizable to the whole US population since patients with some types of public insurance and uninsured patients are not included in the data sources.

## Conclusions

Among NVAF patients who switched from warfarin to a DOAC, risks of stroke/SE and MB varied depending on which DOAC the patient switched to after discontinuing warfarin. Patients who switched to apixaban had lower risks of stroke/SE and MB when compared to those who switched to dabigatran, and similar risk of stroke/SE and lower risk of MB when compared with those who switched to rivaroxaban. These results may help inform clinician decision-making in NVAF patients previously treated with warfarin. Further research is needed to confirm findings of this study.

### Electronic Supplementary Material

Below is the link to the electronic supplementary material.

Supplementary Material 1

## Data Availability

Besides the main data results, additional data will be available in the supplemental materials.
