# Supplementary material for: Effectiveness and safety in non-valvular atrial fibrillation patients switching from warfarin to direct oral anticoagulants in US healthcare claims
Source: J Thromb Thrombolysis. 2024 May 2;57(6):1092–102. doi: 10.1007/s11239-024-02976-1 (PMC11315758; doi:10.1007/s11239-024-02976-1)
Supplement: Supplementary file 1 — Supplementary Material 1 [file 11239_2024_2976_MOESM1_ESM.docx]

## **APPENDICES**

#### **Table A.1** Pre-Propensity Score-Matched Pooled Baseline Characteristics of Patients Switched from Warfarin to Apixaban, Dabigatran, or Rivaroxaban

| Patients who switch from warfarin | Apixaban cohort (reference) | Dabigatran cohort |  | Rivaroxaban cohort |  |
| --- | --- | --- | --- | --- | --- |
|  | N/mean  (%/SD) | N/mean  (%/SD) | STD* | N/Mean  (%/SD) | STD |
| Sample size | 16,553  (100.0%) | 2,738  (100.0%) |  | 14,430  (100.0%) |  |
| Age (years) | 72.77  (11.9) | 70.66  (12.6) | 17.27 | 71.05  (12.5) | 14.12 |
| 18-54 | 967  (5.8%) | 230  (8.4%) | 9.96 | 1,182  (8.2%) | 9.21 |
| 55-64 | 3,080  (18.6%) | 710  (25.9%) | 17.67 | 3,470  (24.1%) | 13.31 |
| 65-74 | 4,769  (28.8%) | 731  (26.7%) | 4.72 | 3,873  (26.8%) | 4.40 |
| 75-79 | 3,375  (20.4%) | 478  (17.5%) | 7.49 | 2,593  (18.0%) | 6.15 |
| ≥80 | 4,362  (26.4%) | 589  (21.5%) | 11.36 | 3,312  (23.0%) | 7.89 |
| Gender | | |  | |  |
| Male | 9,171  (55.4%) | 1,647  (60.2%) | 9.63 | 8,523  (59.1%) | 7.40 |
| Female | 7,382  (44.6%) | 1,091  (39.9%) | 9.63 | 5,907  (40.9%) | 7.40 |
| US geographic region | | |  | |  |
| Northeast | 2,116  (12.8%) | 446  (16.3%) | 9.96 | 2,240  (15.5%) | 7.87 |
| Midwest | 3,453  (20.9%) | 560  (20.5%) | 1.01 | 3,294  (22.8%) | 4.76 |
| South | 7,180  (43.4%) | 1,108  (40.5%) | 5.90 | 5,818  (40.3%) | 6.20 |
| West | 3,744  (22.6%) | 606  (22.1%) | 1.16 | 3,014  (20.9%) | 4.20 |
| Other | 60  (0.4%) | 18  (0.7%) | 4.14 | 64  (0.4%) | 1.28 |
| Baseline comorbidity | | |  | |  |
| Deyo-Charlson comorbidity index | 3.58  (2.9) | 2.78  (2.6) | 28.71 | 2.97  (2.7) | 21.70 |
| CHADS_2_ score | 2.65  (1.4) | 2.48  (1.4) | 12.06 | 2.47  (1.4) | 13.14 |
| 0 | 607  (3.7%) | 153  (5.6%) | 9.15 | 780  (5.4%) | 8.36 |
| 1 | 2,842  (17.2%) | 544  (19.9%) | 6.95 | 3,024  (21.0%) | 9.65 |
| 2 | 4,778  (28.9%) | 818  (29.9%) | 2.22 | 4,180  (29.0%) | 0.23 |
| 3+ | 8,326  (50.3%) | 1,223  (44.7%) | 11.29 | 6,446  (44.7%) | 11.29 |
| CHA_2_DS_2_-VASc score | 4.22  (1.9) | 3.84  (1.9) | 19.82 | 3.86  (1.9) | 18.42 |
| 0 | 256  (1.6%) | 78  (2.9%) | 8.89 | 414  (2.9%) | 9.01 |
| 1 | 1,015  (6.1%) | 250  (9.1%) | 11.31 | 1,283  (8.9%) | 10.48 |
| 2 | 1,851  (11.2%) | 416  (15.2%) | 11.87 | 2,060  (14.3%) | 9.29 |
| 3 | 2,814  (17.0%) | 476  (17.4%) | 1.02 | 2,599  (18.0%) | 2.66 |
| 4+ | 10,617  (64.1%) | 1,518  (55.4%) | 17.81 | 8,074  (56.0%) | 16.77 |
| HAS-BLED score | 3.11  (1.4) | 2.81  1.4 | 22.05 | 2.84  (1.4) | 19.98 |
| 0 | 333.00  (2.0%) | 108.00  (3.9%) | 11.39 | 484.00  (3.4%) | 8.31 |
| 1 | 1,625  (9.8%) | 401  (14.7%) | 14.78 | 1,949  (13.5%) | 11.51 |
| 2 | 3,596  (21.7%) | 663  (24.2%) | 5.92 | 3,614  (25.1%) | 7.85 |
| 3+ | 10,999  (66.5%) | 1,566  (57.2%) | 19.13 | 8,383  (58.1%) | 17.30 |
| Bleeding history | 4,504  (27.2%) | 666  (24.3%) | 6.60 | 3,445  (23.9%) | 7.65 |
| CHF | 4,321  (26.1%) | 638  (23.3%) | 6.50 | 3,283  (22.8%) | 7.81 |
| Diabetes mellitus | 7,064  (42.7%) | 1,130  (41.3%) | 2.84 | 5,920  (41.0%) | 3.34 |
| Hypertension | 14,947  (90.3%) | 2,410  (88.0%) | 7.33 | 12,657  (87.7%) | 8.27 |
| Renal disease | 5,457  (33.0%) | 617  (22.5%) | 23.46 | 3,482  (24.1%) | 19.66 |
| Liver disease | 952  (5.8%) | 148  (5.4%) | 1.51 | 796  (5.5%) | 1.02 |
| Myocardial infarction | 2,261  (13.7%) | 321  (11.72%) | 5.82 | 1,602  (11.1%) | 7.77 |
| Dyspepsia or stomach discomfort | 3,191  (19.3%) | 506  (18.5%) | 2.04 | 2,625  (18.2%) | 2.78 |
| Non-stroke/SE Peripheral vascular disease | 4,852  (29.3%) | 618  (22.6%) | 15.42 | 3,531  (24.5%) | 10.94 |
| Stroke/SE | 2,694  (16.3%) | 428  (15.6%) | 1.76 | 2,039  (14.1%) | 5.98 |
| TIA | 2,316  (14.0%) | 292  (10.7%) | 10.13 | 1,510  (10.5%) | 10.78 |
| Anemia and coagulation defects | 6,196  (37.4%) | 789  (28.8%) | 18.38 | 4,459  (30.9%) | 13.80 |
| Alcoholism | 445  (2.7%) | 74  (2.7%) | 0.09 | 439  (3.0%) | 2.12 |
| Peripheral artery disease | 4,578  (27.7%) | 610  (22.3%) | 12.45 | 3,418  (23.7%) | 9.10 |
| Coronary artery disease | 7,627  (46.1%) | 1,150  (42.0%) | 8.21 | 6,024  (41.8%) | 8.73 |
| Baseline Medication Use |  |  |  |  |  |
| ACE/ARB | 10,418  (62.9%) | 1,795  (65.6%) | 5.47 | 9,017  (62.5%) | 0.93 |
| Amiodarone | 2,303  (13.9%) | 334  (12.2%) | 5.09 | 1,651  (11.4%) | 7.43 |
| Beta blockers | 10,036  (60.6%) | 1,664  (60.8%) | 0.30 | 8,638  (59.9%) | 1.57 |
| H2-receptor antagonist | 1,190  (7.2%) | 183  (6.7%) | 1.99 | 918  (6.4%) | 3.29 |
| Proton pump inhibitor | 5,339  (32.3%) | 842  (30.8%) | 3.23 | 4,323  (30.0%) | 4.96 |
| Statins | 11,196  (67.6%) | 1,768  (64.6%) | 6.48 | 9,393  (65.1%) | 5.39 |
| Anti-platelets | 1,746  (10.6%) | 267  (9.8%) | 2.64 | 1,393  (9.7%) | 2.97 |
| NSAIDS | 2,902  (17.5%) | 534  (19.5%) | 5.08 | 2,698  (18.7%) | 3.03 |
| Dose of the index prescription | | |  | |  |
| Standard dose (5 mg apixaban, 150 mg dabigatran, 20 mg rivaroxaban) | 12,725  (76.9%) | 2,370  (86.6%) | 25.25 | 11,362  (78.7%) | 4.49 |
| Low dose (2.5 mg apixaban, 75 mg dabigatran, 15 mg rivaroxaban) | 3,839  (23.2%) | 359  (13.1%) | 26.38 | 2,664  (18.5%) | 11.67 |
| Other dose (rivaroxaban 10 mg, dabigatran 110 mg) |  | 10  (0.4%) | 8.56 | 430  (3.0%) | 24.78 |
| Events during the baseline | | |  | |  |
| Stroke/SE hospitalization | 1,116  (6.7%) | 175  (6.4%) | 1.41 | 723  (5.0%) | 7.37 |
| Major bleed hospitalization | 826  (5.0%) | 122  (4.5%) | 2.52 | 510  (3.5%) | 7.21 |
| Events during the 90 days before the index date | | |  | |  |
| Stroke/SE | 507  (3.1%) | 76  (2.8%) | 3.94 | 386  (2.7%) | 3.91 |
| Bleeding event | 2,382  (14.4%) | 343  (12.5%) | 5.46 | 1,650  (11.4%) | 8.82 |
| Major bleed hospitalization | 480  (2.9%) | 63  (2.3%) | 2.92 | 278  (1.9%) | 6.21 |
| Gap length between warfarin discontinuation to DOAC initiation | 9  (17.9) | 7  (16.7) | 9.17 | 7  (16.4) | 9.16 |
| Minimum | 1  (0.0%) | 1  (0.0%) | 0.00 | 1  (0.0%) | 0.00 |
| Q1 | 1  (0.0%) | 1  (0.00%) | 0.00 | 1  (0.0%) | 0.00 |
| Median | 1  (0.0%) | 1  (0.00%) | 0.00 | 1  (0.0%) | 0.00 |
| Q3 | 3  (0.0%) | 1  (0.0%) | 0.00 | 1  (0.0%) | 0.00 |
| Maximum | 90  (0.0%) | 90  (0.0%) | 0.00 | 90  (0.0%) | 0.00 |
| Length of warfarin therapy | 267  (317.3) | 175  (207.8) | 34.14 | 221  (267.5) | 15.64 |

*ACE* angiotensin-converting enzyme, *ARB* angiotensin receptor blocker, *CHF* congestive heart failure, *DOAC* direct-acting oral anticoagulant, *NSAID* non-steroidal anti-inflammatory drug, *SD* standard deviation, *SE* systemic embolism, *STD* standard, *TIA* transient ischemic attack, *US* United States

^*^STD difference = 100*[actual STD difference]. STD difference greater than 10 is considered significant.

#### **Table A.2** Pre-Propensity Score-Matched Pooled Outcome Descriptive Table of Patients Switched from Warfarin to Apixaban, Dabigatran, or Rivaroxaban

| Patients who switch from warfarin | Apixaban cohort (reference) | Dabigatran cohort |  | Rivaroxaban cohort |  |
| --- | --- | --- | --- | --- | --- |
|  | N/mean  (%/SD) | N/mean  (%/SD) | STD* | N/mean  (%/SD) | STD |
| Sample size | 16,553  (100.0%) | 2,738  (100.0  %) | 0 | 14,430  (100.0%) | 0 |
| Follow-up time (in days) | 321.69  (330.1) | 292.02  (305.7) | 9.33 | 350.94  (368.5) | 8.36 |
| Minimum | 1 | 1 |  | 1 |  |
| Q1 | 99 | 88 |  | 110 |  |
| Median | 208 | 181 |  | 217 |  |
| Q3 | 414 | 383 |  | 453 |  |
| Maximum | 2163 | 1972 |  | 2294 |  |
| Discontinuation | 8,810  (53.2%) | 1,864  (68.1%) | 30.76 | 8,135  (56.4%) | 6.34 |
| Time-to-discontinuation | 279.61  (273.2) | 289.81  (297.5) | 3.57 | 307.76  (299.3) | 9.82 |
| Switch | 813  (4.9%) | 373  (13.6%) | 30.38 | 1,234  (8.6%) | 14.57 |
| Apixaban | 0  (0.0%) | 114  (30.6%) | 29.47 | 319  (25.9%) | 21.26 |
| Dabigatran | 35  (4.3%) | 0  (0.0%) | 6.51 | 40  (3.2%) | 1.33 |
| Edoxaban | 3  (0.4%) | 0  (0.0%) | 1.90 | 0  (0.0%) | 1.90 |
| Rivaroxaban | 161  (19.8%) | 124  (33.2%) | 21.87 | 0  (0.0%) | 14.02 |
| Warfarin | 614  (75.5%) | 135  (36.2%) | 6.01 | 875  (70.9%) | 10.94 |
| Time-to-switch | 170.74  (191.6) | 234.64  (299.8) | 25.40 | 225.56  (258.0) | 24.12 |
| Disenrollment | 2,608  (15.8%) | 464  (17.0%) | 3.22 | 2,830  (19.6%) | 10.12 |
| Study end | 4,060  (24.5%) | 6  (0.2%) | 79.43 | 1,986  (13.8%) | 27.62 |
| Death | 262  (1.6%) | 31  (1.1%) | 3.89 | 245  (1.7%) | 0.91 |
| Stroke/SE (primary discharge) | 297  (1.8%) | 47  (1.7%) | 0.59 | 260  (1.8%) | 0.06 |
| Hemorrhagic stroke | 38  (0.2%) | 6  (0.2%) | 0.22 | 36  (0.3%) | 0.41 |
| Ischemic stroke | 247  (1.5%) | 39  (1.4%) | 0.57 | 214  (1.5%) | 0.08 |
| SE | 19  (0.1%) | 2  (0.1%) | 1.36 | 15  (0.1%) | 0.33 |
| MB (primary discharge) | 485  (2.9%) | 100  (3.7%) | 4.05 | 646  (4.5%) | 8.20 |
| GI bleeding | 262  (1.6%) | 63  (2.3%) | 5.21 | 395  (2.7%) | 7.95 |
| ICH | 88  (0.5%) | 13  (0.5%) | 0.80 | 75  (0.5%) | 0.16 |
| Other bleeding | 158  (1.0%) | 25  (0.9%) | 0.43 | 218  (1.5%) | 5.04 |
| Stroke/SE time at risk (years) | 0.89  (0.9) | 0.80  (0.9) | 9.16 | 0.97  (1.0) | 8.70 |
| Hemorrhagic stroke | 0.89  (0.9) | 0.81  (0.9) | 9.36 | 0.97  (1.0) | 8.38 |
| Ischemic stroke | 0.89  (0.9) | 0.81  (0.9) | 9.19 | 0.97  (1.0) | 8.68 |
| SE | 0.89  (0.9) | 0.81  (0.9) | 9.26 | 0.97  (1.0) | 8.37 |
| MB time at risk (years) | 0.88  (0.9) | 0.80  (0.8) | 9.36 | 0.96  (1.0) | 7.78 |
| GI bleeding | 0.89  (0.9) | 0.81  (0.9) | 9.36 | 0.97  (1.0) | 8.03 |
| ICH | 0.89  (0.9) | 0.81  (0.9) | 9.44 | 0.97  (1.0) | 8.42 |
| Other bleeding | 0.89  (0.9) | 0.81  (0.9) | 9.17 | 0.97  (1.0) | 8.01 |
| Stroke/SE incidence rate (per 100 person-years) | 2.03 | 2.13 |  | 1.86 |  |
| Hemorrhagic stroke | 0.26 | 0.27 |  | 0.26 |  |
| Ischemic stroke | 1.68 | 1.77 |  | 1.53 |  |
| SE | 0.13 | 0.09 |  | 0.11 |  |
| MB incidence rate (per 100 person-years) | 3.31 | 4.55 |  | 4.66 |  |
| GI bleeding | 1.78 | 2.85 |  | 2.83 |  |
| ICH | 0.60 | 0.59 |  | 0.53 |  |
| Other bleeding | 1.07 | 1.13 |  | 1.56 |  |

*GI* gastrointestinal, *ICH* intracranial hemorrhage, *MB* major bleeding, *SD* standard difference, *SE* systemic embolism, *STD* standard

^*^STD difference = 100*[actual STD difference]. STD difference greater than 10 is considered significant.
